# Sustained Hypoxic Pulmonary Vasoconstriction in the Isolated Perfused Rat Lung: Effect of α_1_-adrenergic Receptor Agonist

**Published:** 2014-05

**Authors:** Farzaneh Ketabchi, Zinab Karimi, Seyed Mostafa S. Moosavi

**Affiliations:** Department of Physiology, School of Medicine, Shiraz University of Medical Sciences, Shiraz, Iran

**Keywords:** Hypoxia, Rat lung, Phenylephrine

## Abstract

**Background:** Alveolar hypoxia induces monophasic pulmonary vasoconstriction in vivo, biphasic vasoconstriction in the isolated pulmonary artery, and controversial responses in the isolated perfused lung. Pulmonary vascular responses to sustained alveolar hypoxia have not been addressed in the isolated perfused rat lung. In this study, we investigated the effect of sustained hypoxic ventilation on pulmonary artery pressure in the present of phenylephrine, an α_1_-receptor agonist, under the above condition.

**Methods: **We performed this study in the isolated perfused rat lung. After preparation, the lungs were divided randomly into five groups of normoxic-normocapnia, hypoxic-normocapnia, phenylephrine pre- or post-treated hypoxic-normocapnia and phenylephrine pre-treated normoxic-normocapnia. Pulmonary hemodynamic, airway pressure and lung weight were measured during 60 min of the experiment for each group.

**Results:** In the phenylephrine-pre-treated hypoxic-normocapnia group we observed a gradual increase in pulmonary artery pressure which approximated the results seen in the phenylephrine-pre-treated normoxic-normocapnia group. In contrast, in the phenylephrine-post-treated hypoxic-normcapnic group, pulmonary artery pressure did not change during the first 3 min of hypoxic-normocapnia. However at 1.5 min after administration of phenylephrine, this pressure began to increase sharply and continued until the end of the experiment. This response was biphasic (0-10 min: acute phase, 10-60 min: sustained phase) with significantly higher pulmonary artery pressure compared to the other groups.

**Conclusion: **This study, for the first time, showed biphasic hypoxic pulmonary vasoconstriction in the isolated perfused rat lung with the sole administration of phenylephrine after but not before hypoxic gas ventilation. This finding suggested a facilitative role of alveolar hypoxia on pulmonary vasoconstriction induced by an α_1_-receptor agonist.

## Introduction

Investigations over several decades have shown that numerous lung diseases and respiratory system disorders may disrupt alveolar ventilation and induce alveolar hypoxia, which may increase pulmonary resistance. This response is known as hypoxic pulmonary vasoconstriction (HPV) which can regulate pulmonary blood flow distribution when it occurs in the local region of the lung, and, pulmonary hypertension during global and persistent alveolar hypoxia. Although HPV has been described since 1946,^1^ its underlying mechanism(s) remain unclear. Many scientists have established in vivo as well as in vitro models to study the mechanism of this physiological response.^2^ The isolated perfused lung is one of the basic methods for determining pulmonary hemodynamic and biochemical events associated with endothelial/epithelial interactions and physiological conditions compared with an in vivo study.^3^^-^^5^ It has been shown that HPV in the rabbit isolated perfused lung and isolated rat artery rings is biphasic with acute and sustained phases.^6^^-^^8^ However, in the isolated perfused rat lung, only short term HPV has been triggered by angiotensin II, EDRF inhibitors, PGF_2α_, and a high concentration of KCl.^9^^-^^12^ No study has reported the effect of sustained hypoxic ventilation in the isolated perfused rat lung.

Phenylephrine (PHE) is an α_1_ and G protein-coupled receptor agonist which causes pulmonary vasoconstriction in an in vivo cat model.^13^^,^^14^ Pre-constriction of pulmonary artery rings in rat with PHE induces a biphasic increase in tension during hypoxia.^7^ In contrast, it has been shown that PHE or norepinephrine do not significantly increase pulmonary artery pressure (PAP) during 3 min of hypoxic ventilation in the isolated perfused rat lung.^10^

By taking the above research into consideration, we aimed to establish, for the first time, biphasic pulmonary vasoconstriction during alveolar hypoxia in the isolated ventilated perfused rat lung. This study was performed in the presence of PHE as a vasoconstrictor for potentiating the hypoxic response of the rat pulmonary vasculature. Interestingly, we noted that addition of PHE to pulmonary circulation only after hypoxic ventilation led to biphasic HPV. 

## Materials and Methods

*Lung Isolation, Perfusion, and Ventilation*

Adult Sprague-Dawley male rats (n=30) were obtained from the Laboratory Animal Breeding Center and used following approval the Ethical Committee for Animal Care, Shiraz University of Medical Sciences. We chose the rat as an experimental model because of its suitable size and accessibility. Additionally, distribution of perfusate flow in the rat lung is approximately uniform compared to larger animals. 

The model of isolated perfused lung was described elsewhere.^3^^-^^5^^,^^9^^,^^15^ Briefly, animals (body weight 250-300 g) were each deeply anesthetized with an i.p. injection of pentobarbital (50 mg/kg body weight) and heparinized (150 U/100 g body weight) for prevention of clot formation during lung preparation. The trachea was cannulated and animals were ventilated with room air (tidal volume 1.2 ml/100 g body weight, respiratory rate 50 beats/min). The chest was opened, after which we cannulated the pulmonary artery and left atrium. The lungs were perfused with 4°C air bubble-free Krebs-Henseleit solution (perfusate) through the pulmonary artery cannula that was connected to a peristaltic pump with a pulsatile flow of 2 ml/min.

The isolated perfused lung was placed in a temperature equilibrated housing chamber and freely suspended from a force transducer for continued monitoring of lung weight. After rinsing the lungs with the perfusate to remove the blood, the perfusion circuit was closed with a total circuit volume of 40 ml. Meanwhile, the flow rate was slowly increased from 2 to 10 ml/min and the entire system (double glass reservoirs, tubing, and housing chamber) was heated from 4°C to 40°C. Concomitantly, the left atrial pressure (LAP) was set at 2-3 cm H_2_O by adjusting the height of venous part of the system to have zone 3 blood flow in the lung. A positive end expiratory pressure (PEEP) of 2 cm H_2_O was chosen for prevention of regional alveolar collapse. Pressures in the pulmonary artery, left atrium, airway, and lung weight were continuously registered by using pressure transducers as well as a force transducer connected to a data acquisition system (Power Lab, AD Instrument, Australia). All lungs included in the study exhibited no signs of hemostasis, edema, or atelectasis, maintained constant mean pulmonary artery and peak airway pressures, and were isogravimetric during the first 30 min of the steady state period. Because flow-rate of the perfusate was constant, changes in PAP were proportional to pulmonary vascular resistance.

*Composition of Ventilatory Gas and Perfusate*

We used two different gas mixtures for lung ventilation: normoxia plus normocapnia (21.0% O_2_ and 5.5% CO_2_ balanced with N_2_) and hypoxia plus normocapnia (1.0% O_2_ and 5.5% CO_2_ balanced with N_2_). The perfusate used for this study contained 120.0 mM NaCl, 1.1 mM K_2_HPO_4_, 1.3 mM MgCl_2_, 4.3 mM KCl, 2.4 mM CaCl_2_, 13.3 mM glucose, and 1 g dextran/100 ml (mw 70000). In all experiments the pH was corrected to normal values by the addition of NaHCO_3_^-^.

*Study Protocol*

At 30 min after surgical preparation (the first 15 min for increasing the flow and temperature, and the second 15 min for stabilizing the preparation), we performed hypoxic ventilation for 10 min to determine the lung's response to alveolar hypoxic-normocapnia in order to ensure a normal vasoreactive response by the pulmonary vessels. Subsequently, hypoxic-normocapnic ventilation was changed to normoxic-normocapnic ventilation for 15 min. Thereafter, we randomly divided the lungs into five experimental groups that included normoxic-normocapnia (NOX, n=7), hypoxic-normocapnia (HOX, n=7), PHE pre- (PHE-HOX, n=5) or post-treated hypoxic-normocapnia (HOX-PHE, n=5) and PHE pre-treated normoxic-normocapnia (PHE-NOX, n=6) in which the lungs were ventilated with normoxic-normocapnic or hypoxic-normocapnic gas for 60 min. In the PHE treated groups, we added PHE (30 µM) to the perfusate 3 min prior or 3 min after the onset of gas ventilation. 

*Statistical Analysis*

Data are given as mean±SEM. Analysis of variance (ANOVA) with the Student-Newman-Keuls (SNK) post hoc test was used for comparison of more than two groups. For comparison of the values during the time course of one group we used the repeated measurement of one-way ANOVA with SNK post hoc. Student's *t *test was used for the comparison of two groups. Significance was assumed when P<0.05.

## Results

We used a gas analyzer (Easy Blood Gas, USA) to measure PO_2_, PCO_2_ and pH of the perfusate for all groups. Table 1 shows these values and the values for osmolarity of the perfusate at 10 min after starting each experiment. These values remained stable during 60 min of experiments.

**Table 1:** PO_2_, PCO_2_, pH, HCO3- and osmolarity in the perfusate during the experimental conditions

	** PO_2_**	** PCO_2_**	** HCO_3_^-^**	**pH**	**Osmolarity**
NOX	97.9±3.05	31.34±1.46	19.71±0.21	7.41±0.02	288.9±2.45
HOX	44±5.31*	29.9±0.58	19.5±0.43	7.42±0.01	290±2.85

Indicates significant difference between hypoxic-normocapnia (HOX, n=17) and  normoxic-normocapnia (NOX, n=13). Values are means±SEM

**Figure 1:**
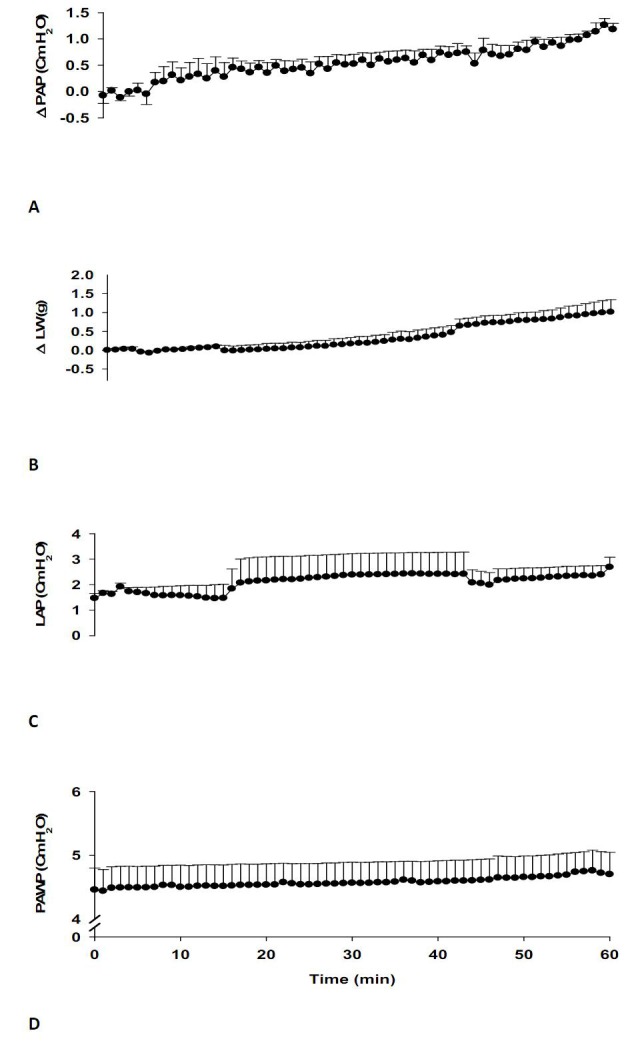
Effects of normoxic-normocapnia (NOX, n=7) ventilation on A: changes in pulmonary artery pressure (∆PAP), B: changes in lung weight (∆LW), C: left atrial pressure (LAP) and D: airway pressure (PAWP). There was no significant alteration between these values during 60 min of the experiment. Values are means±SEM

Baseline values in the NOX group (n=7) were 10.28±1.27 for PAP, 2.47±0.17 for LAP, and 4.46±0.3 cm H_2_O for airway pressures (PAWP). There was no significant alteration between the values of ∆PAP, LAP, PAWP and changes in lung weight (∆LW) during the time course of the experiments (figures 1A-D).

**Figure 2:**
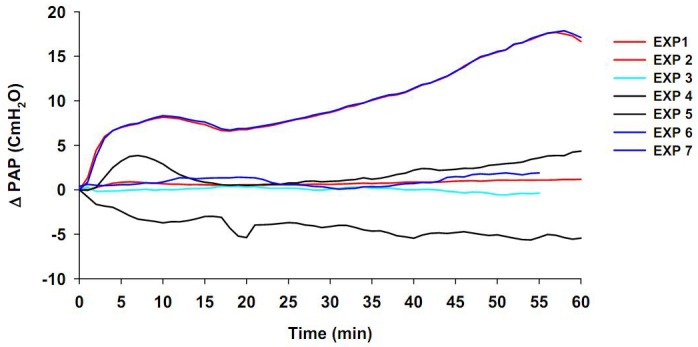
Effects of hypoxic-normocapnia (HOX, n=7) ventilation on A: changes in pulmonary artery pressure (∆PAP) during 60 min of experiments. Ventilation of the lungs with hypoxic-normocapnic gas increased ∆PAP in experiments (EXP) 1, 4 and 6, decreased ∆PAP in EXP 7, or remained unchanged in ∆PAP in EXP 2 and 3. Values are means±SEM

Baseline values in the hypoxic-normocapnic control group (HOX, n=7) were 9.5±1.7 (PAP), 2.65±0.22 (LAP), and 4.96±0.11 cm H_2_O (PAWP). Short term hypoxia increased PAP in only a few experiments (data not shown). Furthermore, with 60 min of ventilation, the lungs with hypoxic-normocapnic gas resulted in increased, decreased or unchanged PAP values (figure 2A). No significant variations were observed in the values of LAP, PAWP and ∆LW during 60 min of the experiment (data not shown).

**Figure 3:**
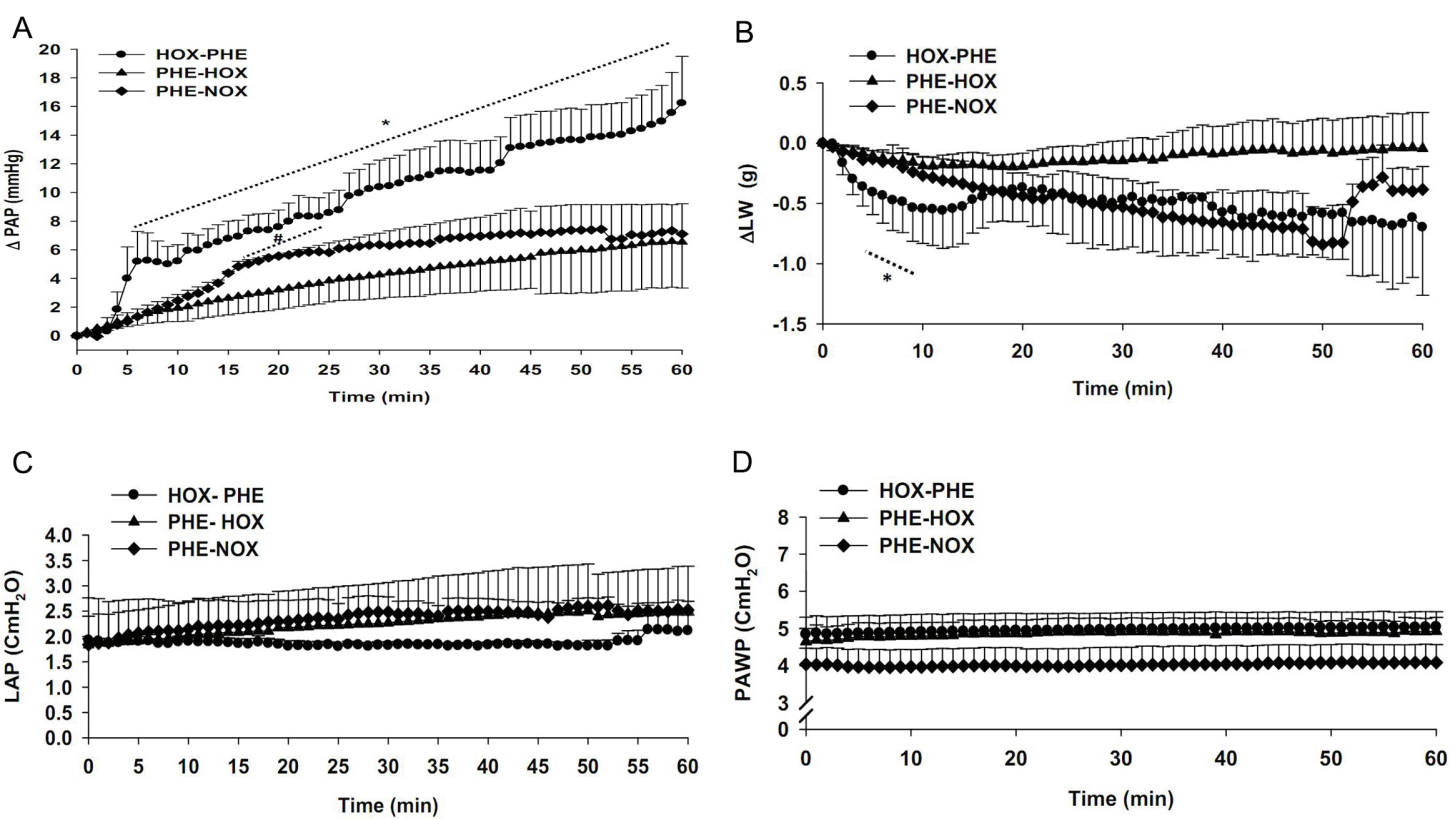
Effects of phenylephrine (PHE) pre-treated normoxic-normocapnic ventilation  (PHE-NOX, n=6, 30 μM, 3 min prior to the experiment), PHE pre-treated hypoxic-normocapnic ventilation  (PHE-HOX, n=5, 30 μM, 3 min prior to the experiment) and PHE post-treated hypoxic-normocapnic ventilation (HOX-PHE, n=5, 30 μM, 3 min after starting the experiment) on A: changes in pulmonary artery pressure (∆PAP), B: changes in lung weight (∆LW), C: left atrial pressure (LAP) and D: airway pressure (PAWP). The HOX-PHE group had significantly higher values for ∆PAP compared to the PHE-HOX and PHE-NOX groups during 6-60 min of the experiment. The values for ∆PAP in the PHE-NOX group were significantly higher than the PHE-HOX group during 17-24 min of the experiments.  All values are means±SEM. *Significant difference (P<0.05) between HOX-PHE and PHE-HOX or PHE-NOX groups; #Significant difference (P<0.05) between PHE-HOX and PHE-NOX groups.

In the PHE (30 μM) pre-treated normoxic-normocapnic group (PHE-NOX, n=6), the baseline values were 10.23±2.17 (PAP), 1.8±0.8 (LAP), and 4.4±0.38 cm H_2_O (PAWP). ∆PAP increased gradually to 7.33±1.47 cm H_2_O at the end of the 60 min period which was significantly higher than its baseline value (figure 3A). There was no significant alteration between the values of LAP, PAWP and ∆LW during time course of experiment in this group (figures 3B-D).

In the PHE pre-treated hypoxic-normocapnia (PHE-HOX, n=5), baseline values were 9.54±2.09 (PAP), 2.1±0.19 (LAP), and 5.68±0.09 (PAWP) cm H_2_O. Ventilation for 60 min the lungs with hypoxic gas resulted in a gradual increase of ∆PAP to 6.54±2.65 at the end of the experiment (figure 3A). We observed no alteration in ∆PAP during most of the experiment between the PHE-HOX and PHE-NOX groups. Interestingly, the values for ∆PAP during 17-24 min of the experiments were significantly higher in the PHE-NOX group compared to the PHE-HOX group. There was no significant difference between LAP, PAWP and ∆LW values during the time course of the experiment between these two groups (figures 3B-D).

In the PHE post-treated hypoxic-normocapnia (HOX-PHE, n=5), baseline values were 10.09±1.8 (PAP), 1.84±0.52 (LAP), and 5.11±0.39 cm H_2_O (PAWP). Hypoxic ventilation did not change PAP during the first 3 min. However 1.5 min after the addition of PHE to the perfusate, ∆PAP increased sharply to 5.24±1.8 at min 7, then decreased to 4.99±1.18 at min 10. Then PAP increased gradually -∆PAP reached 16.23±2.47 at min 60 of the experiment (figure 3A). This response was biphasic (0-10 min: acute phase, 10-60 min: sustained phase). There were significantly higher ∆PAP values during 6-60 min of the experiment compared to the PHE-HOX and PHE-NOX groups. There was no significant alteration between the LAP, PAWP and ∆LW values during the time course of the experiment in this group. Furthermore, ∆LW in the HOX-PHE group was less than the PHE-NOX and PHE-HOX groups however this value was only significant during 4-8 min of the experiments. There was no alteration between the LAP and PAWP values in the PHE-NOX, PHE-HOX and HOX-PHE groups during 60 min of experiments (figures 3B-D).

**Figure 4:**
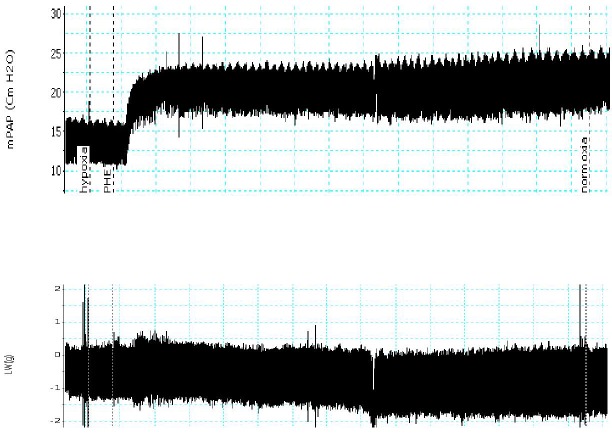
Real tracing that shows the effect of phenylephrine (PHE) on mean pulmonary artery pressure (mPAP) and lung weight (LW) after starting hypoxic ventilation. The sharp increases in mPAP and concomitant decrease in LW are indicated.

Figure 4 shows a real trace of the effect of hypoxic ventilation at the start of PHE on mean PAP (mPAP) and LW. The prominent increase in PAP and concomitant decrease of the lung weight are shown.

## Discussion

The main finding of this study was the observation of a biphasic response by pulmonary vasculature to sustained hypoxic ventilation in the presence of PHE, an α_1_-adrenergic agonist receptor in the isolated perfused rat lung.

Ventilating the lung with normoxic-normocapnic gas did not change PAP, lung weight, airway pressure, LAP, PO_2_, PCO_2_, HCO_3_^-^, pH and osmolarity during steady state and 60 min of the experiment which showed the stability of the isolated perfused lung system in our preparations.

In the PHE treated normoxic-normocapnic group, PAP increased gradually during the time course of the experiment which might be related to increased intracellular Ca^2+ ^concentration after administration of PHE.^13^^,^^14^^,^^16^

In the hypoxic-normocapnia control group, PAP did not increase in all experiments; a result that has been observed in other species.^6^^,^^17^ Hypoxia is reported to elicit a sustained monophasic rise in PAP in vivo, and a biphasic response in the isolated pulmonary artery and isolated perfused lung. Some investigators have shown interspecies variability in the response of pulmonary vessels to alveolar hypoxia.^2^^,^^18^ Although a number of studies have shown sustained HPV in the isolated pulmonary artery in rats, only short term hypoxic response was observed by using high concentrations of KCl, angiotensin II, PGF_2α_ and endothelial derived relaxing factor inhibitors in the isolated artery and isolated perfused rat lung.^9^^-^^12^ In some preparations, researchers exposed the isolated rat artery to anoxic (O_2_=0%) but not hypoxic gas and interpreted the results as a hypoxic response by pulmonary vasculature.^12^

In the present study, PHE sharply increased PAP only after starting hypoxic-normocapnic ventilation. This response was biphasic and approximated the biphasic response of pulmonary vessels to alveolar hypoxia in other species.^17^ Administration of PHE before induction of hypoxia increased PAP similar to the normoxic-normocapnia group. These results might suggest an internal connectivity between the effect of hypoxia and PHE on pulmonary vessels. It has been appreciated that hypoxia enhances intracellular Ca^2+^ in the pulmonary artery which thereby increases PAP. Robertson et al. have shown a relationship of the acute phase of HPV in the isolated rat artery to capacitative Ca^2+^entry from thapsigargin sensitive Ca^2+^ stores and a link between the sustained phase of HPV to influx of Ca^2+^ through voltage independent Ca^2+^ channels.^8^ Hypoxia is believed to inhibit potassium channels which cause membrane depolarization and activation of voltage dependent Ca^2+^ channels.^19^ It has been shown that PHE increases intracellular Ca^2+^ concentration through activation of thapsigargin sensitive Ca^2+^ channels from the endoplasmic reticulum store and non-voltage dependent Ca^2+^ channels.^16^ Since PHE is a sympathetic receptor agonist, the possibility exists of an internal crosstalk between the hypoxia signaling pathway and sympathetic system in vivo. However, this question remains unclear: 'What is the difference between administration of PHE before or after hypoxic gas ventilation?' It is not clear that activation of the sympathetic system or administration of PHE during hypoxia is beneficial. Some scientists have reported that PHE enhances HPV, thereby improving oxygenation in patients with adult respiratory distress syndrome.^20^ On the other hand, it has been shown that PHE does not increase pulmonary vascular resistant and arterial PO_2_.^21^ Many studies are needed to clarify the beneficial effect, if any, of PHE on HPV.

## Conclusion

In this study we established a biphasic HPV in an isolated perfused lung in the present of PHE. This might suggest intracellular connectivity between the mechanisms of PHE and HPV. The beneficial effect of PHE under this condition was unclear.
